# Tedizolid clearance by *in vitro *continuous renal replacement therapy model

**DOI:** 10.1186/cc14193

**Published:** 2015-03-16

**Authors:** SJ Lewis, L Switaj, BA Mueller

**Affiliations:** 1 University of Michigan, Ann Arbor, MI, USA

## Introduction

Tedizolid is an oxazolidinone antibiotic approved to treat acute bacterial skin and soft tissue infection and is under investigation for treatment of nosocomial pneumonia, common in critically ill patients with acute kidney injury. There are limited data on tedizolid disposition in continuous renal replacement therapy (CRRT). This study's purpose was to assess continuous hemofiltration (CHF) and continuous hemodialysis (CHD) influence on tedizolid clearance.

## Methods

Validated, bovine blood-based, *in vitro *CHF and CHD models were used with six new HF 1400 (polysulfone) and six new Multiflow 150 (AN 69) hemodiafilters. Tedizolid's transmembrane clearances (CLTM) during CHF and CHD were assessed by measuring sieving (SC) and saturation (SA) coefficients at various ultrafiltrate (Quf ) (1, 2, 3 l/ hour) and dialysate flow rates (Qd) (1, 2, 3 and 6 l/hour), using a blood flow rate (Qb) of 200 ml/minute. Tedizolid adsorption was tested in a 1 l recirculating CHF model at Quf of 2 l/hour and Qb of 200 ml/minute over 4 hours. Adsorption (%) was calculated after correcting for the dilution by CHF priming volume. Urea was added as a control in all experiments.

## Results

**Figure 1:**
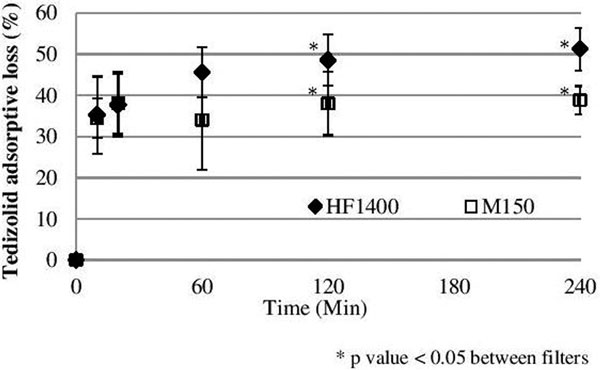


Urea SC and SA were ~1 in all experiments. In CHF, mean tedizolid SC ranged from 0.52 to 0.57 for HF1400 and from 0.50 to 0.54 for M150. CLTM did not differ between filter types for Quf of 1, 2, and 3 l/hour. In CHD, mean tedizolid SA ranged from 0.46 to 0.56 for HF1400 and from 0.38 to 0.44 for M 150. Tedizolid CLTM with the HF1400 was higher than M150 values at Qd of 6 l/hours (*P *< 0.02). Tedizolid exhibited irreversible adsorption within 10 minutes. See Figure 1.

## Conclusion

Tedizolid's CLTM is dependent on hemodiafilter type and Qd for CHD and Quf in CHF. At conventional CRRT rates, tedizolid CLTM appears modest relative to total body clearance and is unlikely to require dose adjustments. CRRT adsorption in the clinical setting is likely less than what we observed in this *in vitro*, continuously recirculating blood model.

